# The new era for the research on the regulation of microorganism-induced inflammation

**DOI:** 10.1186/s41232-024-00359-w

**Published:** 2024-11-12

**Authors:** Kiyoshi Takeda

**Affiliations:** 1 Immunology Frontier Research Center, Osaka University, Osaka, Japan; 2 Department of Microbiology and Immunology, Graduate School of Medicine, Osaka University, Osaka, Japan

Commensal bacteria (microbiota) that inhabit the gastrointestinal tract have been revealed through innovations such as the development of next-generation sequencers and culture techniques in anaerobic conditions. There are more than 10 trillion bacteria and more than 1000 species living in symbiosis in the intestines. It has become clear that commensal bacteria (microbiota) live not only in the gut, but also in various tissues, including the skin. These microbiota are non-self to the immune system, which recognizes and eliminates foreign substances as non-self, but they are not recognized or eliminated by the immune system and live in symbiosis with us. It is becoming clear that these microbiota act on our host and play an extremely important role in maintaining our health. It has become clear that certain microbiota regulate the functions of epithelial cells (epidermal cells), immune cells in the mucosal lining, and other distant tissues. It is also becoming clear that the function of genes of specific microbiota and metabolites produced by microbiota are important as a mechanism of action. Furthermore, it has become clear that an abnormal balance of microbiota (called dysbiosis) is deeply related to the pathogenesis of various diseases. The rapid understanding of the microbiota has been advanced by the analysis of researchers in various research fields such as bacteriology, immunology, informatics, metabolism, and clinical medicine, using their expertise and techniques.

Based on advances in the elucidation of the host-microbiota interactions described above, we gain a better understanding of the regulatory mechanisms of inflammation induced by microorganisms, including infectious diseases. Accordingly, the field of the regulatory mechanisms of inflammation has entered a new era of research.

In this thematic series review, we invited the leading scientists in the research field of the regulation of microbiome-induced inflammation. Dr. Kayama from Osaka University discussed the role of intestinal mesenchymal cells in regulating microbiota-induced intestinal inflammation. Drs. Sugihara and Kamada from Osaka University reviewed the pathogenesis of inflammatory bowel diseases, which pathogenesis is closely linked to gut dysbiosis. They also discussed that the host metabolism is critically involved in the regulation of microbiota composition. Dr. Matsuoka and her colleagues reviewed the skin inflammation caused by the colonization of bacteria on the skin. In particular, they focused on *Staphylococcus aureus* which causes many infectious diseases worldwide. Drs. Yoshinaga and Takeuchi reviewed the regulation of inflammation through mRNA control. They showed that mRNA decay is critically involved in the regulation of inflammation, and this axis is mandatory for resistance to infection with certain viruses.

Finally, I would like to express my sincere appreciation to the scientists who contributed to this special issue. I also hope that this special issue will provide our readers with new knowledge and contribute to the development of their research.

